# The value of privacy for people with dementia

**DOI:** 10.3389/fpsyt.2024.1437813

**Published:** 2025-01-13

**Authors:** Eike Buhr, Mark Schweda

**Affiliations:** Ethics in Medicine, Carl von Ossietzky Universität Oldenburg, Oldenburg, Germany

**Keywords:** privacy, dementia care, well-being, dignity, nursing ethics

## Abstract

**Introduction:**

The concept of privacy marks an astonishing gap in the discussion about care for people with dementia (PwD). In general, questions of privacy play an important role and attract much attention in the ethics of nursing care. Yet, when it comes to dementia care, there is hardly any systematic ethical debate on the topic at all. It almost seems as though PwD lost any plausible interest in privacy and no longer had a private sphere that needed to be considered or protected. However, this not only contradicts widespread moral intuitions but also ignores the views and needs of those affected.

**Arguments:**

This conceptual analysis sets out to explore the value of privacy for PwD. We first outline the origins and dimensions of the concept of privacy itself and point out problems and limitations in the context of dementia. Especially the prevalent liberal conceptions’ dependence on the idea of individual autonomy poses considerable challenges to an adequate understanding of the moral significance of privacy for PwD. Therefore, we subsequently examine alternative ways of conceptualizing the value of privacy in the context of dementia care.

**Conclusion:**

We argue that autonomy-based concepts of privacy may still apply in the early stages of dementia. In the further course of the syndrome, however, the relevance of other normative aspects comes to the fore, especially respect for remaining personal preferences as well as objective criteria of dignity and well-being. Thus, we outline in a differentiated way how and to what extent privacy can be of normative importance even beyond the purview of autonomy and should consequently be considered in dementia care.

## Introduction

In 2017, the daughter of a nursing home resident with dementia filed a complaint with the German Federal Constitutional Court. At the core of the case was the enforcement of her mother’s fundamental rights and the constitutionally guaranteed inviolability of her home. “Are caregivers allowed to just tear open the door to the room?” asked the newspaper *WELT* in a report on the case. “For six years, a daughter has been fighting for privacy for her mother” (1 [own translation]).

This example points to an astonishing gap in the ethical debate about the care of people with dementia (PwD). In general, the topic of “privacy” plays a significant role in the ethics of nursing care. Nursing is considered a sensitive activity that extends into deeply personal areas of other people’s lives. Particularly care involving close physical contacts directly affects the private or intimate sphere of the person being cared for and therefore requires special attentiveness and consideration (2). However, the comprehensive care for the well-being of a person in need of assistance also affects their privacy in many other ways. For example, the growing popularity of outpatient care raises the question of what impact the use of professional care services has on the privacy of the home of those receiving care and their family members (3). And in the setting of the “total institution” (4) nursing home, maintaining the privacy of residents takes on a particularly critical importance (5, 6). Indeed, respect for and protection of the privacy of those being cared for, as well as corresponding duties of restraint, secrecy, and confidentiality also play a significant role in professional ethics standards and codes of the nursing profession (7, 8). In the course of the development of new monitoring and assistance technologies for nursing, the entire topic is currently gaining renewed attention from the perspective of data protection (9, 10).

Remarkably, this intensive ethical discourse on privacy in nursing care seems to fall almost completely silent as soon as the care of PwD is concerned. In this context, only a few scattered comments on the topic can be found (11–13). These mainly address aspects of privacy of family members or professional caregivers that might be affected by ambulatory care in the home setting or by new monitoring technologies (14–18). In contrast, the meaning of privacy for PwD themselves is hardly discussed at all. It could almost appear as though privacy no longer played a significant role for them, as if they lost all comprehensible interest in privacy in the course of their disease and eventually no longer had any private space of their own that needed to be respected or protected in nursing care. Indeed, empirical research shows that the privacy of PwD is frequently violated in the context of care, for example by intrusive behavior or inappropriate familiarity on the part of caregivers (19). An analysis of health apps for this group concluded that more than half of these applications lacked a clear privacy policy (20). This situation is not only difficult to reconcile with the moral conviction that PwD should be recognized as persons to whom we owe respect and consideration (21). It also directly contradicts the views and preferences of those affected themselves as social research shows that PwD consider privacy as an important dimension of their quality of life (22).

Against this backdrop, the present article provides a conceptual analysis of privacy in the context of dementia. We examine to what extent the value, i.e. the moral meaning of privacy for PwD can be made comprehensible and plausible from an ethical point of view. To this end, we first outline the origin and the different dimensions of the concept itself and then demonstrate its difficulties and limitations in the context of dementia. As it turns out, prevailing liberal understandings of privacy have a strong focus on individual autonomy that can be a significant obstacle to an adequate conceptualization of the meaning of privacy for PwD. For this reason, we subsequently explore alternative ways of understanding the “value of privacy” (23) in this context, independent from its function as an expression of the right to individual self-determination (23). While autonomy-based conceptions of privacy may still hold in early stages of dementia, the relevance of recognizable personal preferences as well as objective conditions of dignity and well-being are becoming more important in the further course and advanced stages of the syndrome. In this way, we provide a differentiated analysis of the extent to which privacy is important for people with dementia and how it can be appropriately considered in nursing care across different stages of dementia.

## The liberal notion of privacy and its limits in the context of dementia

Privacy plays an important role in medical and nursing ethics. Its special significance in health-related matters seems to be rooted in the physical closeness and intimacy of medical and nursing practice (24). However, in the form of medical privilege, the confidential handling of health-related information also constitutes a fundamental requirement of the relationship between doctor and patient, nurse and care recipient, as well as researcher and research subject (25).

Similar ideas also play a role in the concept of informational self-determination, which is becoming increasingly important in the wake of digitalization and the emergence of data-intensive medical research and healthcare (26). In the 1960s and 70s, the United States Supreme Court even justified the right of married couples to contraception or women’s right to abortion by recourse to privacy [Griswold v. Connecticut, 381 U.S. 479; 85 Sup. Ct. 1678 (1965), Roe v. Wade, 410 U.S. 113 (1973); 23, 24, 27)]. Although Roe v. Wade was recently revised, this still highlights the importance of privacy as a resource of normative justification. At all these levels, the notion of privacy is closely linked to the claim of non-interference by third parties in one’s bodily concerns and health-related matters and decisions.

The theoretical discourse surrounding the concept of privacy was originally shaped by jurisprudence (28). Here, privacy is traditionally understood as an individual’s right or interest that includes the actively and deliberately exercised control over matters concerning one’s own person (23). Psychological considerations underline that the central concept of control requires both informedness and intentionality (29).

From a moral philosophical point of view, a number of more detailed definitions and distinctions can be made with regard to the function and scope of privacy. The *function* refers to the “value of privacy,” i.e., the purpose that it serves in different areas of life. It provides clues as to why privacy is valued in specific contexts. In the prevailing liberal conceptions, privacy is usually either functionally oriented towards individual autonomy or presupposes a certain degree of autonomy (23, 30). For example, it is seen as a prerequisite for the formation of personal identity as well as for the protection of individual freedom or autonomy (23, 28, 31–33).

The *scope* of privacy indicates the areas of life to which the concept refers. In this context, objects and places as well as knowledge, decisions and actions can be private. Accordingly, privacy can be differentiated in terms of its *decisional*, *informational* and *physical-local* dimensions (23). *Decisional* privacy refers to the possibility of controlling access to one’s personal matters, i.e. being able to decide who has a say in one’s own decisions and actions and who does not (23, 28, 34). The *informational* dimension refers to control over access to information concerning oneself (23, 28, 35–37). *Physical-local* privacy describes control over others’ access to one’s own body, as well as the actively and deliberately exercised regulation of access to one’s places and spaces of living (23, 24).

With regard to PwD, the prevailing liberal conceptions’ focus on autonomy has far-reaching consequences. After all, dementia is accompanied by increasing neurocognitive impairments so that those affected gradually lose capacities usually associated with the ability for self-determination and thus for the active and deliberate exercise of control over their own affairs (38). Indeed, in advanced stages, they may no longer have the explicit notion of an own private sphere and may not even be able to consciously register any violations of this sphere at all.

Accordingly, the claim of PwD to decide on their own personal matters in the sense of *decisional privacy* also seems to be undermined in the course of the disease. In fact, in advanced stages of dementia, relevant decisions are usually taken out of their hands and crucial personal matters are regulated by others on their behalf, for example in the context of legal guardianship and proxy decision-making (39).

Similar observations can be made with regard to the dimension of *informational privacy.* The progressive impairments of short- and long-term memory that accompany dementia mean that those affected increasingly lose the overview of and control over knowledge that concerns their own person, right down to their name, identity and biography. In advanced stages, personal information is therefore usually managed and provided by close third parties (40). In the wake of the development of data-intensive tracking, monitoring, and assistance technologies for PwD, for instance in the field of Ambient Assisted Living, this problem is likely to become even more acute in the future (13, 41).

Finally, comparable trends can also be observed with regard to *physical-local privacy*. Due to their condition, PwD also lose the ability to orient themselves in space and hence to independently control their own living environment. In advanced stages, they can therefore usually neither determine their own place of residence nor provide or deny access to it. Instead, they are cared for at home by family members or professional caregivers, or are placed in nursing facilities (42).

## Perspectives on the value of privacy for people with dementia

Due to changes in cognitive capacities of PwD, theoretical approaches that define privacy in terms of the active and deliberate exercise of control over one’s own affairs are not readily applicable in the context of dementia. However, this does not necessarily mean that privacy is no longer of any moral significance for PwD. After all, the autonomy-based conception of privacy itself could prove to be limited and inadequate in this context. Indeed, PwD explicitly state in surveys that privacy has great significance for their quality of life (22). Values associated with privacy, such as intimacy, confidentiality, social relationships, absence of coercion, are also undoubtedly important in the life and care of PwD (43). Studies in nursing science suggest that even people in advanced stages of dementia do have a sense of privacy that is expressed in their behavior (44–48). Starting from such everyday perspectives and empirical findings, the moral significance of privacy for PwD will be further explored and ethically spelled out in the following sections. In doing so, it becomes apparent that each stage of dementia calls for different lines of argument. Especially with regard to advanced stages, it is crucial to examine to what extent privacy can be conceptualized without recourse to individual autonomy and thus might encompass more than only active and deliberate control over one’s own affairs.

### Early stages: reasserting active control over one’s own affairs

At the beginning of dementia, privacy is of particularly great importance for those affected. In this stage, first memory and orientation problems occur and affect everyday life. Initially, however, this does not derogate the ability to lead a self-determined life. At the same time, knowledge of an increased risk of dementia and especially a diagnosis of dementia constitute highly sensitive personal information that give rise to a strong interest in *informational privacy* (49). In fact, social research indicates that the mere communication of a dementia diagnosis can already lead to increased paternalism and surveillance of those affected by their immediate social environment. For example, one’s decisions are no longer simply accepted but increasingly questioned or even called into doubt. People diagnosed with dementia are no longer readily left to their own devices and find themselves under increased scrutiny and close supervision by others (50). This social reaction can place those affected in a vulnerable position regarding their decisional and physical-local privacy. In addition, the spread of information about someone’s dementia diagnosis can lead to social stigma as well as discrimination, e.g., by employers or insurance companies (51).

Against this backdrop, it appears evident that people in the early stages of dementia have an increased interest in privacy. It is in line with the general autonomy-based reasoning that emphasizes the right to individual self-determination. As the diagnosis of dementia does not per se imply a loss of autonomy, those affected clearly have the right to determine for themselves to which extent they want to involve others in their personal decisions, disclose information about themselves, or allow third parties access to their personal living environment. They are usually also in a position to express and assert this interest in privacy themselves. In fact, studies show that the preservation of their privacy is of particular concern to PwD at these early stages and that its violation causes them distress (47). This could be due to the fact that those affected are often the first ones to notice dementia-related changes, struggle to integrate them into their own self-image, and experience shame and fear of stigma (49). Accordingly, the diagnosis itself, as well as early stages of dementia in general, are associated with various concerns that make the need for privacy immediately plausible. It is therefore particularly important to reassert the right of self-determination of people with beginning dementia as well as their corresponding claims to privacy. This also includes the repudiation of paternalistic tendencies (52).

Notwithstanding this understandable and prima facie undoubtedly justified interest in privacy, however, the diagnosis of (beginning) dementia may give rise to certain moral responsibilities of those affected vis-à-vis third parties. Thus, it could be argued that a diagnosis of dementia can also have far-reaching consequences for life partners or other close relatives that may give them a moral claim to be informed or to have a say (49). For instance, this may pertain to the explanation of changes in the condition and behavior of those affected which can significantly influence their day-to-day interactions with their relatives. If shared professional, financial, or legal interests and concerns will be affected in the future, there may also be a moral responsibility to inform partners or family members of a dementia diagnosis. In particular, the expectation that others will assume care responsibilities may be connected to a moral claim to be informed on the part of the respective individuals (49). However, all these justified interests of third parties do not fundamentally call into question that people with beginning dementia have a right to privacy and to the autonomous regulation of their own affairs. At most, they may correspond to moral responsibilities that must be weighed against this right in specific cases.

### Middle stages: respect for personal identity and subjective preferences

As their condition advances, PwD become increasingly dependent on assistance. The progressive impairment of cognitive abilities affects executive functions and hence also activities of daily living, such as choosing suitable clothing or preparing meals. In particular, the impairment of language and judgment skills compromises the ability to process complex information and to make well-considered self-determined decisions. This can also lead to behavior that is dangerous to oneself or others, for example at home or in traffic. Despite this successive diminishment of autonomy, however, a sense of privacy and de facto preferences with regard to privacy can still be observed in PwD at this stage. This raises the question to what extent the moral meaning of privacy can be made explicit without reference to personal autonomy.

Social research shows that PwD continue to be concerned about privacy even as their condition becomes more severe. In fact, the very awareness of the progression of their dementia and the experience of the symptoms described seem to induce an increased desire for intimacy and familiarity, i.e. privacy (22). Apart from verbal statements, this interest in privacy can also become manifest in corresponding behavior. For example, PwD often show defensive reactions when doctors attempt to perform examinations without advance notice or consultation (46). The (non-verbal) rejection of unsolicited nursing measures and the feigning of sleep to avoid interactions with caregivers and other nursing home residents can also be regarded as expressions of a claim to decisional privacy. The ostentatious deviation from caregivers’ suggestions may be interpreted as an attempt to assert a say in one’s own daily schedule and thus also as a desire for decisional privacy. Furthermore, the possibility to have an undisturbed conversation and talk about intimate fears and concerns in familiar surroundings is a frequently expressed need of PwD that points to a desire for informational privacy (12). Finally, behaviors such as choosing a particular place to sit (46), furnishing one’s room with personal items (45) and the “embodied memory” expressed this way (53), or the frequently described desire to “go home” (54) can also underscore a concern to maintain some form of *physical-local privacy* (22, 47). Especially for women with dementia, the importance of one’s own handbag and the contents stored in it as “biographical objects” may serve as another example (55).

It may no longer be possible to interpret such privacy-related behaviors of people in the middle stage of dementia as expressions of personal autonomy in a sophisticated moral philosophical sense. Nevertheless, it would hardly appear acceptable to simply dismiss them as morally irrelevant or summarily disregard them without careful consideration. Eventually, they seem to represent physical and habitual expressions of deeply rooted personal priorities and preferences regarding one’s own lifestyle and relationships with others. Acknowledging and respecting them can therefore be crucially important for the personal identity and subjective well-being of those affected. In this vein, it could be argued that privacy-related behaviors, even if not fully autonomous, still carry moral significance in the middle stages of dementia, particularly when they can be interpreted as expressions of fundamental or identity-relevant needs, wishes, or feelings. With regard to the aspect of personal identity, such behaviors may represent certain characteristic traits of the person concerned that deserve respect, especially if we accept the idea of an “embodied self” (56) of PwD that becomes apparent in their physical appearance and habitualized demeanor. With regard to subjective well-being, one could speak of “experiential interests” (57) of PwD regarding privacy. In contrast to so-called “critical interests,” that is, well-considered judgments formed in light of personal values and life plans, “experiential interests” rather refer to immediate, momentary experiences in the present. Although people in the middle stage of dementia are sometimes no longer able to make decisions based on “critical interests,” such “experiential interests” must still be respected and considered as far as possible because their violation would be detrimental to their well-being or even cause them harm.

Against this backdrop, respecting the privacy-related preferences of people in the middle stages of dementia would require the consideration of statements and behaviors of those affected that may no longer qualify as expressions of autonomous, informed and well-considered judgements.^1^ Of course, such an approach raises considerable hermeneutic and moral-practical questions and poses challenges in the context of nursing care. Thus, the interpretation of erratic utterances or nonverbal behavior usually does not provide clear, unambiguous directives for concrete care provision. In many cases, it would probably remain ultimately indeterminable which statements and behaviors of PwD could be regarded as manifestations of a specific desire for privacy, at all. Furthermore, privacy-related behavior also does not provide any clues as to what moral relevance should be assigned to the desire for privacy vis-a-vis other preferences of the person concerned or requirements of their well-being, such as personal hygiene, safety and protection against self-harm. Therefore, the question of how to deal with verbally or non-verbally expressed privacy preferences in practice would eventually be hard to decide.

### Advanced dementia: objective conditions of dignity and well-being

In late stages of dementia, preference-based arguments to explain the value of privacy for PwD also reach their limits. The progredient impairment of language increasingly restricts the possibility of communicating subjective preferences. In advanced stages, the behavior of those affected eventually also becomes more erratic and difficult to interpret. Ultimately, the condition affects the underlying cognitive categories and mental operations. As a consequence, the abstract concept of privacy, as well as the subjective awareness of one’s own private sphere and its violations, may be lost. The diminishment of a sense of shame and social appropriateness, e.g., in connection with clothing, food intake, or excretion, could be interpreted in this vein (59).

Under these conditions, ethical approaches aiming to establish the moral significance of privacy for individuals with advanced dementia must ultimately rely on aspects other than the perspectives of those affected themselves. Such approaches could be termed objectivistic since they do not refer to the subjective views, attitudes, and evaluations of the individuals directly concerned. At first sight, this seems to be at odds with the normative principles of the modern liberal understanding of morality and its ethical reflection in categories of individual autonomy and self-determination (60). Nevertheless, there exist at least a number of argumentative precedents for such an objectivistic exploration of the value of privacy for people with advanced dementia.

A first starting point could be the concept of human dignity (61). In the Basic Law for the Federal Republic of Germany, respect for dignity is more fundamental than the individual right to self-determination and the free development of the personality. Accordingly, prominent court rulings derived and enforced the legal prohibition of self-deprecation through self-display or self-degradation, for example, in cases about peep shows or “dwarf tossing” (BVerwGE 64, 274; NVwZ 1993). Along the lines of this form of paternalism aimed at protecting human dignity, one could argue that the privacy of people in advanced stages of dementia should be protected in order to prevent them from self-deprecation. Such paternalistic protection of privacy may even appear more justifiable in this case as it refers to an involuntary self-deprecation and would not override an autonomous will. However, the concept of dignity is itself notoriously ambiguous and controversial. Approaches that see dignity as grounded in certain capacities, such as autonomy, reach their limits in the context of advanced dementia, just like corresponding autonomy-based understandings of privacy (62). Concepts of human dignity based on cosmological or theological considerations may be able to circumvent these difficulties but are based on particular religious or ideological presuppositions that are not generally shared in modern pluralistic societies (62). Moreover, the question arises as to whether protecting individuals with dementia from self-deprecation only aims to preserve their dignity or is actually more about upholding their esteem or remembrance in the eyes of third parties like partners or family members. These concerns are all the more serious as such protection from self-deprecation might require measures that could conflict with the current will and subjective well-being of PwD, for example, the prevention of physical intimacy in socially inadequate situations. For this reason, the importance of privacy in the context of dementia is sometimes relativized in the ethical discussion. As the historical development of the concept is entangled with “the repression of physicality by rational reason”, privacy can appear to “be of secondary importance for the quality of life of people with advanced dementia [ … ] (e.g., compared to social proximity)” (63 [own translation]).

Arguments that focus on the best interest of the persons concerned and thus ultimately on objective preconditions of their well-being could provide an alternative. In the sense of weak paternalism, one could argue that privacy is a necessary condition for the well-being of people with advanced dementia in certain respects and contexts, and therefore should be protected even if they themselves have lost any discernible subjective interest in it. In this sense, it could be argued that the careless disclosure of personal information may enable abuse or even criminal activities and pose serious risks to the physical well-being or financial security of the individuals concerned (13). Similarly, physical-local and informational privacy may constitute a necessary precondition for the development of personal care relationships that are fundamental to the well-being of those affected. If PwD benefit from the care of persons who feel close and connected to them and responsible for them (even if they themselves may no longer recognize these persons at all), then conditions that enable and strengthen such caring closeness, attachment, and responsibility should be protected (64). Finally, respect for the informational and physical-local privacy of people with advanced dementia may also provide protection against forms of objectification and instrumentalization that are detrimental to their well-being, such as public display and humiliation or sexual exploitation. Of course, such notions of trans-subjective preconditions of individual well-being ultimately presuppose an objective theory of the good life and hence also take on considerable theoretical burdens of justification. Moreover, some objective approaches such as Nussbaum’s anthropologically grounded list of fundamental human capacities have been criticized for not granting persons with cognitive impairments the possibility of a life that can count as fully human (65). Finally, such approaches also touch upon the difficult problem of how to balance the protection of the objective well-being of those affected with their momentary subjective impulses or preferences in cases of conflict (66).

## Conclusion

In contemporary ethical discussions about the central importance of privacy in nursing care, the perspective of PwD finds virtually no systematic consideration. It almost seems as if the principle no longer played any particular role in their lives and in their care. The fact that their privacy is actually often undermined in practice and has to be defended against various violations seems to confirm the power of such a view. It raises the question of the value of privacy for PwD, which becomes even more important in the face of the emergence of new data-intensive tracking, monitoring, and assistance technologies (67).

As argued here, this desideratum is probably not least related to the specific theoretical implications of prevailing liberal concepts of privacy. These concepts understand privacy primarily in terms of an active and deliberate control over one’s own affairs and thus presuppose autonomy or are functionally oriented toward it. However, as their condition progresses, PwD lose the ability to take charge of their own affairs in an active and deliberate way. In order to make the meaning of privacy intelligible in this context, we therefore need a more encompassing understanding of the concept that is not exclusively based on autonomy.

By reference to the typical stages of dementia, we have explored possibilities for justifying the moral meaning and function of privacy for PwD. It has become apparent that each stage requires different lines of argument. In early stages, the liberal autonomy-based understanding of privacy is still relevant since those affected are generally capable of managing their own affairs in a self-determined manner. However, as cognitive abilities such as speech and judgment become more impaired in middle stages, the autonomy-based concept of privacy reaches its limits. Nevertheless, it is still possible to identify an interest of PwD in privacy based on a range of verbal and nonverbal behaviors. This interest should be recognized and protected in order to support their embodied sense of self and their subjective well-being. Of course, it becomes increasingly difficult for outsiders to assess the privacy preferences of those affected and to balance them against the increased need for care and protection. In advanced stages of dementia, we must therefore find other ways of substantiating the moral significance of privacy for PwD. One option are objectivist arguments that make the meaning of privacy for people with advanced dementia plausible without referring to the perspective of those affected themselves, for example, by recourse to ideas of dignity or human flourishing. The associated burdens of justification may be considerable. Yet, in the interest of the protection of privacy even in late stages of dementia, they should not be evaded. For example, objective notions of a good life postulating basic human capabilities could be used to defend privacy as the basis for the ability to maintain caring relationships that are beneficial to PwD.

Further conceptual, empirical and normative research is needed to better understand the meaning of privacy for PwD. First, the views of affected persons themselves should be considered in more detail and in a more differentiated manner in order to find out what needs, emotions, and moral concerns privacy comprises for them. Of course, prevailing theoretical conceptions of privacy seem hardly suitable for such a socio-empirical exploration, given their narrow focus on the idea of individual autonomy. Before we can explore the value of privacy for PwD in empirical studies, we therefore need to develop a more comprehensive understanding of the concept itself and its various implications, connotations, and references to related notions such as closeness, intimacy, or security. For this purpose, it is possible to draw on values and functions associated with privacy as well as on established criteria in the care of PwD. By interweaving this conceptual-philosophical analysis with empirical studies, for example in the form of qualitative social research with affected people and relatives, an empirically informed concept of privacy could be developed. Such a concept would have at least two advantages: First, it would provide a basis for making the meaning and value of privacy for PwD systematically plausible. This could help raise the awareness of caregivers for privacy-relevant behavior of PwD. Thus, even those who are no longer in a position to decide for themselves who they want to grant access to their own room, such as the nursing home resident mentioned at the beginning of this contribution, may well have an understandable and legitimate interest in protection, security, and familiar close relationships – in short, in privacy. Moreover, a perspective developed through empirically informed ethical discussions in the context of dementia could also help to overcome the narrow focus on the liberal principle of individual self-determination and expand the general academic debate about privacy as such. Eventually, this could contribute to the further illumination of the multifaceted and morally complex nature of privacy, even beyond the field of dementia care.

## Data Availability

The original contributions presented in the study are included in the article/**Supplementary Material**. Further inquiries can be directed to the corresponding author.

## Footnotes

1 Richard Dworkin and Rebecca Dresser discuss the scope of non-autonomous decisions. Cf. Dworkin (57); Dresser (58).
