# Supplementary material for: The value of privacy for people with dementia
Source: Front Psychiatry. 2025 Jan 13;15:1437813. doi: 10.3389/fpsyt.2024.1437813 (PMC11770680; doi:10.3389/fpsyt.2024.1437813)
Supplement: Supplementary file 1 [file DataSheet1.pdf]

Ethik Med (2022) 34:591–607
https://doi.org/10.1007/s00481-022-00723-9

ORIGINALARBEIT

Der Wert des Privaten für Menschen mit Demenz

Eike Buhr · Mark Schweda

Eingegangen: 3. Mai 2022 / Angenommen: 4. August 2022 / Online publiziert: 26. Oktober 2022
© Der/die Autor(en) 2022

Zusammenfassung Der Begriff der Privatheit markiert eine erstaunliche Leerstelle in der Diskussion um die Pflege von Menschen mit Demenz (MmD). Der sonst intensiv geführte pflegeethische Diskurs über Fragen der Privatheit scheint hier nahezu vollständig zu verstummen, so als verlören MmD im Verlauf ihrer Erkrankung jedes nachvollziehbare Interesse an einer Privatsphäre und verfügten über keinerlei privaten Bereich mehr, den man bei ihrer pflegerischen Versorgung beachten oder schützen müsste. Eine solche Vorstellung widerspricht allerdings nicht nur verbreiteten moralischen Intuitionen, sondern auch den Auffassungen und Bedürfnissen der Betroffenen selbst. Vor diesem Hintergrund gehen wir der Frage nach, inwieweit sich die Bedeutung von Privatheit für MmD ethisch verständlich und plausibel machen lässt. Zu diesem Zweck werden zunächst die Herkunft und die verschiedenen Bedeutungsdimensionen des Privatheitsbegriffs selbst umrissen, um anschließend seine Schwierigkeiten und Grenzen im Kontext demenzieller Erkrankungen aufzuzeigen. Wie sich dabei herausstellt, kann insbesondere der ausgeprägte Autonomiebezug vorherrschender liberaler Privatheitskonzepte ein erhebliches Hindernis für eine angemessene Konzeptualisierung der Bedeutung der Privatheit für MmD darstellen. Aus diesem Grund loten wir im Anschluss unterschiedliche Möglichkeiten aus, wie sich der „Wert des Privaten“ im Kontext demenzieller Erkrankungen auch losgelöst vom Recht auf individuelle Selbstbestimmung konzeptualisieren ließe. Während autonomiebasierte Konzepte von Privatheit in frühen Stadien noch tragen mögen, wird mit Blick auf den weiteren Krankheitsverlauf daher auch der Relevanz von erkennbaren persönlichen Präferenzen sowie objektiven Bedingungen von Würde und Wohlergehen nachgegangen. Auf diesem Weg lässt sich differenziert aufzei-

Eike Buhr (✉) · Prof. Dr. Mark Schweda
Abteilung Ethik in der Medizin, Carl-von-Ossietzky Universität Oldenburg, Ammerländer Heerstraße 114–118, 26129 Oldenburg, Deutschland
E-Mail: eike.buhr@uni-oldenburg.de

gen, inwiefern Privatheit auch für MmD von Bedeutung sein und im pflegerischen Umgang mit ihnen angemessen berücksichtigt werden kann.

Schlüsselwörter Privatheit · Demenz · Wohlergehen · Würde · Pflegeethik

The value of privacy for people with dementia

Abstract
Definition of the problem The concept of privacy has been astonishingly absent in the discussion about dementia care. In general, questions of privacy receive a lot of attention in nursing ethics; however, when it comes to dementia care, hardly any systematic ethical debate on the topic can be found. It almost seems as though people with dementia had lost any comprehensible interest in privacy and no longer had any private sphere that needed to be considered or protected. However, this not only contradicts widespread moral intuitions but also ignores the views and needs of those affected.
Arguments We first outline the origins and dimensions of the concept of privacy and point out problems and limitations in the context of dementia. Especially the prevalent liberal conceptions' dependence on the idea of autonomy poses significant challenges to an adequate conceptualization of the moral significance of privacy for people with dementia. Therefore, we subsequently explore alternative ways of conceptualizing the "value of privacy" in the context of dementia care.
Conclusion Autonomy-based concepts of privacy may still be valid in the early stages of dementia. However, in the further course of the syndrome, the relevance of other normative aspects comes to the fore, especially respect for remaining personal preferences as well as objective criteria of dignity and well-being. Thus, we outline in a differentiated manner how and to what extent privacy can be of normative importance beyond the purview of autonomy and should consequently be taken into consideration in dementia care.

Keywords Privacy · Dementia · Well-being · Dignity · Nursing ethics

Einleitung

Im Sommer 2017 reichte die Tochter einer demenziell erkrankten älteren Pflegeheimbewohnerin im Rheinland nach einer längeren juristischen Auseinandersetzung mit dem Heimbetreiber Beschwerde beim Bundesverfassungsgericht ein. Im Kern ging es um die Durchsetzung des von der Klägerin stellvertretend wahrgenommenen Hausrechts ihrer Mutter und die verfassungsmäßig zugesicherte Unverletzlichkeit ihrer Wohnung. „Dürfen die Pfleger einfach so die Tür zum Zimmer aufreißen?", fragte die WELT in einem Bericht über den Fall. „Seit sechs Jahren streitet eine Tochter für ihre demenzkranke Mutter um Privatsphäre" (Dowideit 2017).

Das Beispiel verweist auf eine erstaunliche Leerstelle in der ethischen Auseinandersetzung um die Pflege von Menschen mit Demenz (MmD). Allgemein spielt das Thema „Privatheit" im pflegeethischen Diskurs eine durchaus bedeutsame Rolle.

Pflege gilt als eine sensible Tätigkeit, die in zutiefst persönliche Lebensbereiche anderer hineinreicht. Gerade körpernahe pflegerische Verrichtungen berühren die Privat- oder Intimsphäre der zu Pflegenden unmittelbar und verlangen daher besondere Achtsamkeit und Rücksichtnahme (Immenschuh 2018). Doch auch sonst betrifft die in der Pflege zum Tragen kommende umfassende Sorge für das Wohlergehen einer hilfsbedürftigen Person deren private Belange auf vielfältige Weisen. So wirft die wachsende Verbreitung ambulanter Pflege die Frage auf, welche Auswirkungen der Einsatz professioneller Pflegedienste auf das häusliche Privatleben der Gepflegten und ihrer Familienangehörigen hat (Dyck et al. 2005). Und im stationären Setting der „totalen Institution“ (Goffman 1961) Pflegeheim erhält die Wahrung der Privatsphäre der Bewohnenden eine besonders kritische Bedeutung (Behr et al. 2013; Schell 2011). Tatsächlich spielen der Respekt und Schutz der Privatsphäre der zu Pflegenden und entsprechende Zurückhaltungs-, Verschwiegenheits- und Vertraulichkeitspflichten auch eine maßgebliche Rolle in professionsethischen Standards und Kodizes des Pflegeberufs (ICN 2014; DGF 2013). Im Zuge der Entwicklung neuer Monitoring- und Assistenztechnologien für die Pflege erhält die gesamte Thematik gegenwärtig unter dem Aspekt des Datenschutzes erneut verstärkt Aufmerksamkeit (Alkhatib et al. 2019, 2021).

Bemerkenswerterweise scheint dieser intensiv geführte ethische Diskurs um Privatheit in der Pflege nahezu vollständig zu verstummen, sobald es um die pflegerische Versorgung von MmD geht. In diesem Kontext finden sich lediglich verstreut einige spärliche Bemerkungen zum Thema (O’Brolcháin und Gordijn 2019; Digby und Bloomer 2014; Zwijsen et al. 2011). Dabei werden hauptsächlich Aspekte der Privatheit von Angehörigen oder professionell Pflegenden angesprochen, die beispielsweise durch die ambulante Versorgung im häuslichen Umfeld oder durch neue technische Monitoringsysteme tangiert sein könnten (Jacobsson und Davidsson 2015; Eltis 2005; Francis und Francis 2017; Welsh et al. 2003; Carlson 2001). Eine grundlegende Auseinandersetzung mit der Bedeutung von Privatheit für MmD sucht man hingegen vergebens. Fast könnte der Anschein entstehen, Privatheit spiele für sie selbst keine nennenswerte Rolle mehr, so als verlören sie im Verlauf ihrer Erkrankung jegliches nachvollziehbare Interesse an einer Privatsphäre und verfügten irgendwann über keinerlei eigenen privaten Bereich mehr, den man bei ihrer pflegerischen Versorgung beachten oder schützen müsste. Tatsächlich zeigen empirische Untersuchungen, dass MmD in der Pflege häufig Verletzungen ihrer Privatsphäre wie aufdringlichem Verhalten oder unangemessenen Vertraulichkeiten ausgesetzt sind (van der Geugten und Goossensen 2020). Und eine Analyse von Gesundheitsapps für diese Personengruppe kam vor einigen Jahren zu dem Ergebnis, dass in mehr als der Hälfte der Fälle eine klare Datenschutzbestimmung fehlte (Rosenfeld et al. 2017). Diese Ausgangslage ist nicht nur schwer vereinbar mit der heute allgemein akzeptierten moralischen Überzeugung, dass auch MmD als vollwertige Personen anzuerkennen sind, denen wir entsprechend Respekt und Achtung schulden (Schmidhuber 2013). Sie widerspricht auch unmittelbar den Auffassungen und Bedürfnissen von Betroffenen selbst. So konnte etwa in sozialwissenschaftlichen Befragungen gezeigt werden, dass Privatheit aus Sicht von MmD durchaus eine wichtige Dimension ihrer eigenen Lebensqualität darstellt (Dichter et al. 2016).

Vor diesem Hintergrund wendet sich der vorliegende Beitrag dem Begriff der Privatheit im Kontext von Demenz zu. Dabei gehen wir der leitenden Fragestellung nach, inwieweit sich der Wert, also die moralische Bedeutung von Privatheit für MmD ethisch verständlich und plausibel machen lässt. Zu diesem Zweck werden zunächst die Herkunft und die verschiedenen Bedeutungsdimensionen des Begriffes selbst umrissen, um anschließend seine Schwierigkeiten und Grenzen im Kontext demenzieller Erkrankungen aufzuzeigen. Wie sich dabei herausstellt, kann insbesondere der ausgeprägte Autonomiebezug vorherrschender liberaler Privatheitsverständnisse ein erhebliches Hindernis für eine angemessene Konzeptualisierung der Bedeutung von Privatheit für MmD darstellen. Aus diesem Grund loten wir im Anschluss explorativ unterschiedliche Möglichkeiten aus, den „Wert des Privaten“ (Rössler 2001) in diesem Zusammenhang auch losgelöst von seiner Funktion als Ausdruck des Rechts auf individuelle Selbstbestimmung verständlich zu machen. Während autonomiebasierte Konzeptionen von Privatheit in frühen Stadien demenzieller Erkrankungen noch tragen, wird mit Blick auf den weiteren Krankheitsverlauf und fortgeschrittene Stadien auch der Relevanz von erkennbaren persönlichen Präferenzen sowie objektiven Bedingungen von Würde und Wohlergehen nachzugehen sein. Auf diesem Weg lässt sich in differenzierter Weise aufzeigen, inwiefern Privatheit auch für MmD von Bedeutung sein und im pflegerischen Umgang mit ihnen angemessen berücksichtigt werden kann.

Der liberale Begriff der Privatheit und seine Grenzen im Kontext der Demenz

Privatheit spielt eine wichtige Rolle im medizin- und pflegeethischen Diskurs. Dabei scheint ihre besondere Bedeutung in gesundheitlichen Angelegenheiten zunächst in der Körpernähe und Intimität ärztlicher und pflegerischer Tätigkeiten begründet zu sein (Allen 2014). Doch auch der vertrauliche Umgang mit gesundheitsbezogenen Informationen stellt eine grundlegende Anforderung an die Beziehungen zwischen medizinischem Personal und Patientinnen und Patienten, zwischen Pflegenden und Gepflegten sowie zwischen medizinisch Forschenden und Studienteilnehmenden dar (Currie 2005).

Dieser Grundsatz der Vertraulichkeit findet etwa Ausdruck in der ärztlichen Schweigepflicht. Auch im Gedanken der informationellen Selbstbestimmung, der im Zuge der Digitalisierung und des Aufkommens datenintensiver medizinischer Forschung und Gesundheitsversorgung zunehmend an Bedeutung gewinnt, spielen ähnliche Vorstellungen eine Rolle (Deutscher Ethikrat 2020). Der Oberste Gerichtshof der Vereinigten Staaten hat in den 1960er und 70er-Jahren darüber hinaus sogar das Recht verheirateter Paare zur Verhütung oder das Recht auf Abtreibung unter Rekurs auf Privatheit begründet (Griswold v. Connecticut, 381 U.S. 479; 85 Sup. Ct. 1678 (1965), Roe v. Wade, 410 U.S. 113 (1973); Allen 2014; DeCew 1997; Rössler 2001). Auch wenn das betreffende Urteil zur Abtreibung jüngst revidiert wurde, verdeutlicht dies die Bedeutung von Privatheit als normativer Begründungsressource. Allgemein ist der Gedanke der Privatheit auf all diesen Ebenen eng mit dem

Anspruch der Nichteinmischung Dritter in die eigenen körperlichen Belange und gesundheitlichen Angelegenheiten und Entscheidungen verknüpft.

Der theoretische Diskurs um den Begriff der Privatheit ist ursprünglich von den Rechtswissenschaften geprägt (Schoeman 1992). Dort wird Privatheit herkömmlicherweise als ein individuelles Recht oder ein Interesse aufgefasst, das die Beschränkung des Zugangs zur eigenen Person beziehungsweise die aktiv und bewusst ausgeübte Kontrolle über ihre Belange umfasst (Rössler 2001). Aus moralphilosophischer Perspektive lassen sich dabei sowohl mit Blick auf die Funktion des Privaten als auch hinsichtlich seines Umfangs eine Reihe näherer Bestimmungen und Unterscheidungen vornehmen.

Die *Funktion* bezieht sich auf den „Wert des Privaten", also den Zweck, den Privatheit in unterschiedlichen Lebensbereichen erfüllt. Sie liefert damit Hinweise darauf, warum Privatheit in distinkten Kontexten wertgeschätzt wird. Dabei ist Privatheit in den vorherrschenden liberalen Konzeptionen in der Regel entweder funktional auf Autonomie ausgerichtet oder setzt ein bestimmtes Maß an Autonomie voraus (Rössler 2001; Bloustein 1964). So wird sie unter anderem als Bedingung für den Schutz von Freiheit oder (personaler) Autonomie sowie die Ausbildung personaler Identität betrachtet (Allen 1988; Schoeman 1992; Reiman 1976, S. 37; Cohen 2002, S. 57; Rössler 2001). Auch der in der Konzeption von Rössler essentielle Begriff der Kontrolle erfordert aus handlungspsychologischer Sicht sowohl Informiertheit als auch Intentionalität (Oesterreich 1981, S. 24 ff.).

Der *Umfang* des Privaten gibt an, auf welche Lebensbereiche sich Privatheit bezieht. Dabei können sowohl Gegenstände und Orte als auch Wissen oder Entscheidungen bzw. Handlungen privat sein. Dementsprechend lässt sich Privatheit in Anlehnung an Rössler (2001) hinsichtlich ihrer *dezisionalen, informationellen* sowie *physisch-lokalen* Dimension differenzieren. Die *dezisionale* Privatheit bezeichnet die Möglichkeit, den Zugang zur eigenen Person zu kontrollieren, also entscheiden zu können, wem hinsichtlich der eigenen Handlungen und Entscheidungen ein Mitspracherecht eingeräumt wird und wem nicht (Rössler 2001, S. 25, 114 f.; Schoeman 1992, S. 22; Gavison 1980, S. 423). Im Zentrum der *informationellen* Dimension steht die Kontrolle über den Zugang zu Informationen, die die eigene Person betreffen (Westin 1967; Fried 1968; Schoeman 1992; Moore 2003; Rössler 2001). Die *physisch-lokale* Privatheit wiederum beschreibt zunächst die Kontrolle über den Zugriff anderer auf den eigenen Körper und daran anknüpfend dann auch die aktiv und bewusst ausgeübte Regulation des Zugangs zu den Orten und Räumen des eigenen Wohnens (Allen 2014; Rössler 2001).

Im Hinblick auf MmD hat der im Zusammenhang mit Funktion und Umfang der Privatheit deutlich werdende Autonomiebezug der vorherrschenden liberalen Konzeptionen weitreichende Konsequenzen. Schließlich gehen demenzielle Erkrankungen mit einer Zunahme schwerer neurokognitiver Beeinträchtigungen einher, in deren Verlauf die Betroffenen die Fähigkeit zur Selbstbestimmung und damit zur bewusst und aktiv ausgeübten Kontrolle über die eigenen Angelegenheiten nach und nach einbüßen (Jungert 2018). Tatsächlich mögen sie in fortgeschrittenen Stadien nicht einmal mehr über die Vorstellung einer eigenen Privatsphäre verfügen und etwaige Verletzungen einer solchen Sphäre auch überhaupt nicht mehr bewusst registrieren können. Entsprechend scheint auch der Anspruch von MmD, im Sinne

der *dezisionalen Privatheit* selbst über ihre persönlichen Belange zu entscheiden, im Verlauf der Erkrankung mehr und mehr an Boden zu verlieren. Tatsächlich werden ihnen in fortgeschrittenen Krankheitsstadien relevante Entscheidungen weitgehend abgenommen und grundlegende persönliche Angelegenheiten etwa im Rahmen einer rechtlichen Betreuung stellvertretend durch andere geregelt (wobei Patientenverfügungen immerhin eine Möglichkeit bieten, die dezisionale Privatheit der Betroffenen in gewissem Umfang zu wahren) (Denke 2011). Ähnliches lässt sich auch im Hinblick auf die Dimension der *informationellen Privatheit* beobachten. Die mit demenziellen Erkrankungen einhergehenden fortschreitenden Beeinträchtigungen des Kurz- und Langzeitgedächtnisses führen dazu, dass Betroffene zunehmend den Überblick und die Kontrolle über die sie selbst betreffenden Wissensbestände verlieren, bis hin zum eigenen Namen und der persönlichen Identität und Lebensgeschichte. Personenbezogene Informationen werden daher in fortgeschrittenen Stadien meist von nahestehenden Dritten verwaltet und entsprechende Auskünfte durch sie autoritativ erteilt (Lind 2005). Im Zeichen der Entwicklung datenintensiver Ortungs-, Überwachungs- und Assistenztechnologien für die Pflege von MmD, etwa im Bereich Ambient Assisted Living, dürfte sich diese Problematik künftig noch weiter verschärfen (O'Brolcháin und Gordijn 2019; Welsch und Buhr 2022). Schließlich sind vergleichbare Tendenzen auch mit Blick auf die *physisch-lokale Privatheit* festzustellen. MmD büßen auf Grund ihrer Erkrankung ebenfalls die Fähigkeit zur räumlichen Orientierung und damit auch zur eigenständigen Kontrolle ihres eigenen Lebensumfelds ein. In fortgeschrittenen Stadien können sie infolgedessen meist weder den eigenen Aufenthaltsort noch den Zugang zu ihm uneingeschränkt selbst bestimmen, sondern werden etwa durch familial bzw. professionell Pflegende häuslich betreut oder in einer Pflegeeinrichtung untergebracht (Knoll et al. 2017).

Der Wert der Privatheit für Menschen mit Demenz

Theoretisch-konzeptionelle Ansätze, die Privatheit im Sinne einer bewusst ausgeübten aktiven Kontrolle über die eigenen Belange definieren, lassen sich im Kontext demenzieller Erkrankungen nicht ohne Weiteres anwenden. Das bedeutet allerdings keineswegs zwingend, dass Privatheit für MmD tatsächlich keinerlei Bedeutung mehr hat. Vielmehr könnte sich auch die besagte autonomiebasierte Konzeption von Privatheit selbst in diesem Zusammenhang als verkürzt und unzulänglich erweisen. In der Tat messen MmD Privatheit in Befragungen ausdrücklich eine wichtige Bedeutung für ihre Lebensqualität bei (Dichter et al. 2016). Auch mit Privatheit assoziierte Werte wie Intimität, Wahrnehmung sozialer Beziehungen, Vertraulichkeit, Abwesenheit von Zwang sind in der Pflege, Versorgung und Lebenswelt von MmD zweifellos von Bedeutung (Smebye und Kirkevold 2013). Tatsächlich legen pflegewissenschaftliche Studien nahe, dass selbst Betroffene in fortgeschrittenen Stadien durchaus ein Gefühl für Privatheit besitzen, das sich z.B. in ihrem Verhalten äußert (Pirhonen und Pietilä 2015; Graneheim et al. 2001; McColgan 2005; Shenk et al. 2004; Dröes et al. 2006). Ausgehend von derartigen lebensweltlichen Perspektiven und empirischen Befunden soll die moralische Bedeutung von Privatheit für MmD im Folgenden weiter exploriert und ethisch ausbuchstabiert werden. Dabei

zeigt sich, dass mit Blick auf die verschiedenen Phasen demenzieller Erkrankungen jeweils andere Argumentationsmuster in den Vordergrund treten. Gerade mit Blick auf weiter fortgeschrittene Stadien wird zu erörtern sein, inwieweit Privatheit auch unabhängig von Autonomie konzeptualisiert werden kann und damit mehr als die aktive Kontrolle der eigenen Angelegenheiten einschließt.

Beginnende Demenz: Die Bekräftigung der aktiven Kontrolle über eigene Belange

Gerade zu Beginn demenzieller Erkrankungen kommt Privatheit für MmD eine wichtige Bedeutung zu. Krankheitsbedingt treten hier bereits erste Beeinträchtigungen der Gedächtnisleistung und des Orientierungsvermögens auf, die sich auf den Alltag der Betroffenen auswirken, allerdings zunächst noch nicht die Fähigkeit zu einer selbstbestimmten Lebensführung in Frage stellen. Dabei stellen schon das Wissen um ein erhöhtes Risiko für eine demenzielle Erkrankung und erst recht die Diagnose einer beginnenden Demenz zugleich hochgradig sensible persönliche Informationen dar, die ein starkes Interesse an *informationeller Privatheit* begründen (Herbst 2021). Tatsächlich deuten sozialwissenschaftliche Untersuchungen darauf hin, dass bereits das Bekanntwerden der bloßen Demenzdiagnose zur verstärkten Bevormundung und Überwachung der Betroffenen durch das nähere soziale Umfeld führen und in der Folge auch ihre *dezisionale und physisch-lokale Privatheit* beeinträchtigen kann. Die eigenen Entscheidungen werden nicht mehr mit der gleichen Selbstverständlichkeit akzeptiert, sondern verstärkt hinterfragt oder gar in Zweifel gezogen. Menschen mit einer diagnostizierten Demenz werden nicht mehr ohne Weiteres sich selbst überlassen, sondern stehen gleichsam unter verschärfter Beobachtung und engmaschigerer Beaufsichtigung durch andere (Beard und Fox 2008). Darüber hinaus kann das Bekanntwerden einer Demenzdiagnose auch zur gesellschaftlichen Stigmatisierung sowie zur Diskriminierung führen, etwa durch Arbeitgeber oder Versicherungsunternehmen (Schweda et al. 2018).

Vor diesem Hintergrund erscheint das Interesse von MmD an Privatheit in frühen Stadien demenzieller Erkrankungen unmittelbar nachvollziehbar. Dabei lässt es sich hier wie bei allen anderen Personen auch zunächst ohne Weiteres gemäß der autonomiebasierten Argumentation unter Bezugnahme auf ihr Recht auf individuelle Selbstbestimmung verständlich machen. Die Diagnose einer beginnenden Demenz bedeutet nicht augenblicklich den Verlust der Autonomiefähigkeit. Entsprechend haben die Betroffenen prima facie auch ohne Zweifel das Recht, selbst zu bestimmen, inwieweit sie andere in ihre persönlichen Entscheidungen einbeziehen, Informationen über sich preisgeben oder Dritten Zugang zu ihrem persönlichen Lebensumfeld einräumen. Dabei sind sie in aller Regel auch durchaus in der Lage, dieses Privatheitsinteresse selbst zum Ausdruck zu bringen und ihm Geltung zu verschaffen. Tatsächlich äußern MmD in diesem frühen Stadium in sozialwissenschaftlichen Befragungen, dass ihnen gerade die Wahrung ihrer Privatsphäre besonders am Herzen liegt bzw. ihre Verletzung ihnen zu schaffen macht (Dröes et al. 2006). Dies könnte darauf zurückzuführen sein, dass Betroffene demenziell bedingte Veränderungen oft zunächst selbst bemerken. Ihre Integration in das eigene Selbstbild stellt eine Herausforderung dar und ist oftmals auch mit Scham verbunden (Herbst 2021).

Entsprechend sind gerade die Diagnose und das Frühstadium mit diversen Ängsten verbunden, die das Bedürfnis nach der Schutzfunktion des Privaten begründen. Angesichts der angesprochenen Tendenzen im sozialen Umfeld kommt es entsprechend vor allem darauf an, das Selbstbestimmungsrecht von Menschen mit einer beginnenden Demenz und ihren darin gründenden Anspruch auf Privatheit zu bekräftigen und Ansätze zur fürsorglichen Bevormundung und Überwachung in die Schranken zu weisen (Deutscher Ethikrat 2012).

Ungeachtet ihres nachvollziehbaren und prima facie zweifellos berechtigten Interesses an Privatheit mögen sich aus der Diagnose einer (beginnenden) Demenz für die Betroffenen allerdings gewisse moralische Verantwortlichkeiten gegenüber Dritten ergeben, die dieses Recht auf Privatsphäre begrenzen können. So lässt sich argumentieren, dass eine Demenzdiagnose auch für Lebenspartnerinnen und -partner oder andere nähere Angehörige weitreichende Konsequenzen haben kann, die einen Anspruch auf Mitteilung oder Mitsprache begründen können (Herbst 2021). Das betrifft beispielsweise die Erklärung möglicher Veränderungen im Befinden und Verhalten der Betroffenen sowie im alltäglichen Zusammenleben mit ihnen. Auch sofern perspektivisch gemeinsame Angelegenheiten und Planungen, etwa beruflicher, finanzieller oder juristischer Art, tangiert sind, kann sich eine moralische Verantwortung zur Mitteilung einer Demenzdiagnose an Lebenspartner oder Familienmitglieder ergeben. Schließlich mag insbesondere die Erwartung einer künftigen Übernahme von Sorgeverantwortung einen moralischen Anspruch auf frühzeitige Einweihung der betreffenden Personen rechtfertigen (Herbst 2021). All diese berechtigten Interessen Dritter an der Mitteilung einer Demenzdiagnose, vermögen allerdings den Anspruch von Menschen mit einer beginnenden Demenz auf ihre Privatsphäre und die selbstbestimmte Regelung ihrer eigenen Angelegenheiten keinesfalls grundsätzlich in Frage zu stellen. Sie machen allenfalls moralische Verantwortlichkeiten geltend, die punktuell gegen diesen Anspruch abgewogen werden müssen und ihn im Ergebnis auch einschränken können.

Mittlere Stadien: Respekt vor erkennbaren Präferenzen

Im mittleren Erkrankungsstadium sind MmD zunehmend auf Hilfe angewiesen. Die fortschreitende Beeinträchtigung kognitiver Fähigkeiten betrifft etwa Exekutivfunktionen, was sich beispielsweise im Verlust alltagspraktischer Fähigkeiten wie der zur Auswahl passender Kleidung oder der Zubereitung von Mahlzeiten äußert. Die weitere Schwächung des Sprach- und Urteilsvermögens schränkt insbesondere die Fähigkeit ein, komplexere Informationen zu verarbeiten und auf dieser Grundlage wohlerwogene selbstbestimmte Entscheidungen zu treffen. Dadurch kann es vermehrt auch zu selbst- oder fremdgefährdendem Verhalten kommen, etwa im Haushalt oder im Straßenverkehr. Trotz dieses fortschreitenden Verlustes der Autonomiefähigkeit im engeren Sinne lassen sich bei MmD de facto allerdings auch weiterhin Bedürfnisse nach Privatheit, Empfindungen von Privatheit und Präferenzen hinsichtlich Privatheit ausmachen. Damit drängt sich zunehmend die bereits angesprochene Frage auf, inwieweit sich die moralische Bedeutung von Privatheit in diesem Kontext auch unabhängig von autonomiebasierten Deutungen des Konzepts verständlich machen lässt.

Befragungen von MmD zeigen, dass ihnen auch im Fortschreiten ihrer Erkrankung weiterhin an Privatheit gelegen ist. Tatsächlich scheint gerade die Erkenntnis der Progredienz der eigenen Krankheit mit den beschriebenen Symptomen sogar vielfach mit einem erhöhten Bedarf an Intimität und Vertrautheit, das heißt an Privatheit einherzugehen (Dichter et al. 2016). Dieses Privatheitsinteresse lässt sich auch über explizite sprachliche Stellungnahmen hinaus an entsprechenden Verhaltensweisen ablesen. So sind etwa körperliche Abwehrreaktionen gegenüber Ärztinnen und Ärzten zu beobachten, die ohne Ankündigung bzw. Absprache eine Untersuchung vornehmen (McColgan 2005). Auch die Ablehnung ungefragter pflegerischer Maßnahmen sowie das Vortäuschen von Schlaf, um Interaktionen mit Pflegenden und anderen Heimbewohnern zu umgehen, lassen sich als Ausdruck *dezisionaler Privatheit* deuten. Das ostentative Abweichen von Vorschlägen Pflegender mag ebenfalls als Versuch zu interpretieren sein, eine Mitsprache bei der eigenen Tagesgestaltung und damit ein Verlangen nach dezisionaler Privatheit geltend zu machen. Darüber hinaus ist die Möglichkeit, ungestört ein Gespräch zu führen und in vertrauter Umgebung über intime Ängste und Sorgen zu sprechen, ein oftmals geäußertes Bedürfnis von MmD, das auf einen Wunsch nach *informationeller Privatheit* verweist (Digby und Bloomer 2014, S. 39). Verhaltensweisen wie die Auswahl eines bestimmten Sitzplatzes (McColgan 2005), die Einrichtung des eigenen Zimmers mit persönlichen Gegenständen (Shenk et al. 2004) und das so zum Ausdruck gebrachte „embodied memory" (Schües 2020, S. 103) oder das Verlangen, „nach Hause zu gehen" (Dekkers 2011, S. 292 f.), mögen schließlich auch das Anliegen unterstreichen, eine Form *physisch-lokaler Privatheit* aufrecht zu erhalten (Dröes et al. 2006; Dichter et al. 2016). Als ein weiteres Beispiel insbesondere bei Frauen mit Demenz kann die Bedeutung der eigenen Handtasche und des in ihr aufbewahrten Inhaltes als „biographische Objekte" dienen (Buse und Twigg 2014, S. 17).

Derartige privatheitsbezogene Äußerungen und Verhaltensweisen von Menschen im mittleren Stadium einer demenziellen Erkrankung mögen nicht mehr in jedem Fall als Ausdruck des Anliegens einer im vollen Sinne autonomen Kontrolle und Regelung der eigenen Angelegenheiten aufzufassen sein. Gleichwohl wird man sie kaum einfach ohne Weiteres als moralisch vollkommen bedeutungslose und unerhebliche Erscheinungen abtun oder sich gar kurzerhand über sie hinwegsetzen wollen. Immerhin scheinen hier auf einer körperlichen bzw. habituellen Ebene, tief verwurzelte persönliche Präferenzen und Prioritäten bezüglich der eigenen Lebensweise und des Verhältnisses zu anderen Menschen im näheren Lebensumfeld zum Tragen zu kommen, die für die personale Identität und das subjektive Wohlergehen der Betroffenen von erheblicher Bedeutung sein können. In diesem Sinne ließe sich argumentieren, dass auch die nicht vollständig autonomen privatheitsbezogenen Willensäußerungen von Menschen im mittleren Stadium einer demenziellen Erkrankung moralisch durchaus ins Gewicht fallen, sofern sie als Manifestationen entsprechender grundlegender oder gar identitätsrelevanter Bedürfnisse, Wünsche oder Empfindungen aufgefasst werden können. Mit Blick auf das subjektive Wohlergehen ließe sich von „experiential interests" (Dworkin 1994, S. 201) von MmD im Bezug auf ihre Privatheit sprechen, also Interessen, die sich eher auf das momentane Erleben in der Gegenwart beziehen und nicht im Sinne von „critical interests" als Ausdruck wohlüberlegter, im Lichte persönlicher Wertvorstellungen und Lebens-

pläne formulierter Anliegen zu begreifen sind. Da Menschen mit fortgeschrittener Demenz teilweise nicht mehr dazu in der Lage sind, Entscheidungen vor dem Hintergrund ihrer „critical interests“ zu treffen, gilt es solche „experiental interests“ im Umgang mit ihnen so weit wie möglich zu achten und zu berücksichtigen, weil ihre Verletzung ihrem Wohl abträglich wäre oder ihnen sogar Schaden zufügen würde. Mit Blick auf den Aspekt der Identität mögen sich in derartigen privatheitsbezogenen Verhaltensweisen überdies auch gewisse charakteristische Persönlichkeitszüge der Betroffenen wiedererkennen lassen, die es auch in ihrer aktuellen Verfassung im Sinne eines „verkörperten Selbst“ (Fuchs 2018) weiterhin zu respektieren gilt.

Um die Privatheitspräferenzen von Menschen mit fortgeschrittener Demenz zu berücksichtigen, kann und muss demnach auch Äußerungen und Verhaltensweisen der Betroffenen Rechnung getragen werden, die nicht mehr unbedingt als Ausdruck einer autonomen, mithin informierten und wohlerwogenen Willensbildung gelten können.[1] Allerdings wirft eine solche Vorgehensweise in der Versorgungpraxis zugleich erhebliche hermeneutische wie auch moralisch-praktische Fragen und Schwierigkeiten auf. Zum einen sind die Wünsche und Willensäußerungen von MmD nämlich mit dem Fortschreiten der Erkrankung für Außenstehende in der Regel immer schwerer nachvollziehbar. Insbesondere aus nonverbalen Äußerungen lassen sich nicht mehr ohne Weiteres Schlussfolgerungen für die konkrete Ausgestaltung der Pflege und Versorgung ziehen. Letztlich bliebe aus diesen Gründen vermutlich vielfach unklar, welche Verhaltensweisen von MmD überhaupt als Manifestationen von bestimmten Privatheitswünschen anzusehen wären. Zum anderen lässt sich selbst aus einem manifest gegebenen Privatheitsverhalten nicht ableiten, welches moralische Gewicht dem fraglichen Wunsch nach Privatheit gegenüber anderen Präferenzen der Betroffenen oder Erfordernissen ihres Wohlergehens wie etwa der Köperhygiene und insbesondere ihrer Sicherheit und dem Schutz vor Selbstgefährdung beizumessen wäre. Hier bliebe in der Folge letzten Endes die Frage nach dem konkreten praktischen Umgang mit verbal oder nonverbal zum Ausdruck gebrachten Privatheitspräferenzen offen.

Weit fortgeschrittene Demenz: objektive Bedingungen von Würde und Wohlergehen

In späten Stadien demenzieller Erkrankungen stoßen auch präferenzbasierte Argumente zur Erläuterung des Wertes von Privatheit für MmD an Grenzen. Die Beeinträchtigung der Sprachfähigkeit schränkt die Möglichkeit zur Mitteilung subjektiver Präferenzen bereits im mittleren Stadium zunehmend ein. Doch auch das Verhalten der Betroffenen wird schließlich erratischer und schwerer zu deuten. Letztlich zieht die Erkrankung auch die zu Grunde liegenden Gehalte und begrifflich-kategorialen Ordnungsleistungen des Bewusstseins selbst in Mitleidenschaft. In der Folge mögen auch der abstrakte Begriff von Privatheit sowie der subjektive Sinn für die eigene Privatsphäre und ihre Verletzungen verloren gehen, worauf etwa der Verlust von

[1] Über die Reichweite nicht-autonomer Entscheidungen diskutieren grundlegend Richard Dworkin und Rebecca Dresser. Vgl. Dworkin (1994); Dresser (1995).

Schamgefühl, z.B. im Zusammenhang mit Bekleidung, Nahrungsaufnahme oder Ausscheidung, hindeutet (Cipriani et al. 2016).

Unter diesen Bedingungen einer weit fortgeschrittenen demenziellen Erkrankung müssen ethische Argumentationsansätze, die die moralische Bedeutung von Privatheit für MmD auch weiterhin plausibel machen sollen, letzten Endes offenkundig auf andere Bezugsgrößen als die Perspektive der betroffenen Personen selbst rekurrieren. Solche in diesem Sinne objektiven, also nicht von den subjektiven Ansichten, Einstellungen und Bewertungen der unmittelbar betroffenen Individuen abhängigen Argumentationsstrategien scheinen zunächst in einem gewissen Spannungsverhältnis zu den normativen Grundsätzen des modernen liberalen Moralverständnisses und seiner ethischen Reflexion in Kategorien individueller Autonomie zu stehen (Lillehammer 2012). Gleichwohl lassen sich auch in diesem Bereich zumindest eine Reihe argumentativer Präzedenzen aufzeigen, an die eine Exploration des Wertes von Privatheit für Menschen mit fortgeschrittener Demenz anknüpfen könnte.

Einen ersten Anknüpfungspunkt bietet etwa die grundlegende Stellung des Menschenwürdegedankens im deutschen Verfassungsrecht (Trescher 2015). Das Gebot der Achtung der Menschenwürde ist im Grundgesetz für die Bundesrepublik Deutschland selbst dem individuellen Recht auf freie Entfaltung der Persönlichkeit normativ vor- und übergeordnet. Daraus ist anknüpfend an die kantische Moralphilosophie unter anderem auch ein rechtliches Verbot der Selbstentwürdigung durch Selbstzurschaustellung oder Selbsterniedrigung abgeleitet worden, das in prominenten Gerichtsurteilen zu Peepshows oder „Zwergenwurf" zur Geltung gebracht wurde (BVerwGE 64, 274; NVwZ 1993). In Anlehnung an einen derartigen „verfassungsrechtlichen Würdepaternalismus" könnte auch mit Blick auf Menschen in fortgeschrittenen Stadien einer demenziellen Erkrankung argumentiert werden, dass ihre Privatheit zu schützen sei, um sie vor Selbstentwürdigung zu bewahren. Es ließe sich sogar ins Feld führen, dass ein solcher paternalistischer Privatheitsschutz in diesem Fall noch eher zu vertreten sei, da es sich schließlich um eine unwillkürliche Selbstentwürdigung handle und man sich nicht über einen autonomen Willen hinwegsetzen würde. Allerdings ist der Würdebegriff seinerseits notorisch vieldeutig und kontrovers. Ansätze, die Würde in bestimmten Fähigkeiten wie der zur Autonomie begründet sehen, geraten im Kontext fortgeschrittener Demenz ebenso an Grenzen wie entsprechende autonomiebasierte Privatheitsverständnisse (Thiele 2013). Kosmologisch oder schöpfungstheologisch begründete Würdebegriffe mögen diese Schwierigkeiten zwar umgehen können, beruhen aber auf religiösen oder weltanschaulichen Voraussetzungen, die in modernen pluralistischen Gesellschaften nicht allgemein geteilt werden (Thiele 2013). Überdies stellt sich mit Blick auf den Schutz von MmD vor Selbstentwürdigung die Frage, inwiefern hier tatsächlich die Würde der Betroffenen selbst oder eher ihr Ansehen bzw. Andenken in den Augen Dritter geschützt würde. Diese Bedenken wiegen umso schwerer, als ein solcher Schutz vor Selbstentwürdigung möglicherweise Maßnahmen erforderlich machen könnte, die mit dem aktualen Willen und subjektiven Wohlbefinden der Betroffenen in Konflikt geraten können, beispielsweise die Unterbindung von körperlicher Nähe in sozial inadäquaten Situationen. Aus diesem Grund wird die Bedeutung von Privatheit im Kontext demenzieller Erkrankungen in der ethischen Diskussion mitunter relativiert: „Privatsphäre als historisch entstandenes Konzept" beruhe „zentral auf

dem Zurückdrängen von Körperlichkeit durch rationale Vernunft“, sodass sie „[f]ür die Lebensqualität von Menschen mit weit fortgeschrittener Demenz [...] ggf. von untergeordneter Bedeutung (z.B. im Vergleich zu sozialer Nähe)“ (Klie et al. 2010, S. 7) sei.

Eine Alternative zu einem solchen ethischen Würde- und Privatheitspaternalismus wird durch Argumentationen eröffnet, die auf das beste Interesse der Betroffenen und damit letztlich auf die objektiven Bedingungen ihres Wohlergehens abheben. So könnte man etwa im Sinne eines schwachen Paternalismus die Auffassung vertreten, dass Privatheit in bestimmten Hinsichten und Zusammenhängen eine notwendige Bedingung für das Wohlergehen von Menschen mit weit fortgeschrittener Demenz bildet und darum auch dann zu schützen ist, wenn diese selbst jedes erkennbare subjektive Interesse daran verloren haben. In diesem Sinne ließe sich beispielsweise argumentieren, dass die unbedarfte Preisgabe bestimmter personenbezogener Informationen missbräuchliche oder sogar kriminelle Verwendungen ermöglichen und in der Folge mit Gefahren für das leibliche Wohl oder für die finanzielle Sicherheit der Betroffenen einhergehen kann (O'Brolcháin und Gordijn 2019). In ähnlicher Weise mögen physisch-lokale und informationelle Privatheit eine notwendige Voraussetzung für die Ausbildung und Aufrechterhaltung bestimmter für das Wohlergehen der Betroffenen grundlegender Formen persönlicher Sorgebeziehungen bilden. Wenn für das Wohlergehen von MmD auch die Sorge von Personen zuträglich ist, die sich ihnen nah und verbunden und für sie verantwortlich fühlen (auch wenn sie selbst diese Personen überhaupt nicht mehr erkennen mögen), dann sind Bedingungen zu schützen, die solche sorgende Nähe, Bindung und Verantwortungsübernahme ermöglichen und stärken[2]. Schließlich mag die Achtung der informationellen und physisch-lokalen Privatsphäre von Menschen mit einer weit fortgeschrittenen Demenz auch einen Schutz vor Formen der Objektivierung und Instrumentalisierung bieten, die ihrem Wohlergehen abträglich sind, etwa durch öffentliche Zurschaustellung und Erniedrigung oder sexuelle Ausbeutung. Freilich setzen derartige Vorstellungen von subjektunabhängigen Bedingungen individuellen Wohlergehens letzten Endes eine objektive Theorie des guten Lebens voraus und nehmen damit durchaus ebenfalls erhebliche theoretische Begründungslasten auf sich. Erschwerend kommt hinzu, dass gerade objektive Ansätze wie Nussbaums anthropologisch fundierte Liste fundamentaler menschlicher Befähigungen verschiedentlich dafür kritisiert wurden, Personen mit kognitiven Beeinträchtigungen nicht die Möglichkeit eines im vollen Sinne menschlichen Lebens zuzugestehen (Kittay und Carlson 2010). Schließlich berühren solche Ansätze auch das gerade im Kontext demenzieller Erkrankungen viel diskutierte diffizile Problem, wie im Konfliktfall zwischen dem Schutz des objektiven Wohls der Betroffenen und ihren momentanen subjektiven Impulsen oder Präferenzen abzuwägen ist (Deutscher Ethikrat 2018).

[2] Ausführlicher zu diesem Aspekt: Buhr und Schweda (2023)

Schluss

In zeitgenössischen ethischen Diskussionen um die zentrale Bedeutung von Privatheit in der Pflege findet die Perspektive von MmD kaum systematisch Berücksichtigung. Fast scheint es, als spielte das Prinzip in ihrem Leben und ihrer pflegerischen Versorgung keinerlei besondere Rolle mehr. Dass ihre Privatsphäre de facto auch in der Praxis immer wieder in Frage gestellt wird und gegen Grenzverletzungen verteidigt werden muss, scheint die Wirkmacht einer solchen Sichtweise zu bestätigen und wirft die Frage nach dem Wert von Privatheit für MmD auf, die gerade angesichts des Aufkommens neuer datenintensiver Tracking-, Monitoring- und Assistenztechnologien an Brisanz und Dringlichkeit gewinnt (Schweda und Schicktanz 2021).

Wie sich zeigte, dürfte das angesprochene Desiderat nicht zuletzt mit dem begrifflich-theoretischen Zuschnitt vorherrschender liberaler Privatheitskonzepte zusammenhängen. Diese fassen Privatheit vornehmlich im Sinne der aktiven und bewussten Kontrolle über die eigenen Angelegenheiten und setzen insofern Autonomiefähigkeit voraus oder sind doch funktional auf sie ausgerichtet. MmD verlieren jedoch im Zuge des Fortschreitens ihrer Erkrankung die Fähigkeit zur aktiven und bewussten Wahrnehmung der eigenen Belange. Um die Bedeutung von Privatheit in diesem Kontext verständlich zu machen, ist daher ein weiteres, nicht ausschließlich autonomiebasiertes Verständnis des Begriffs ins Auge zu fassen.

Orientiert an typischen Stadien des Krankheitsverlaufs haben wir Möglichkeiten zur Begründung der ethischen Bedeutung und Funktion von Privatheit für MmD exploriert. Dabei zeigte sich, dass hier je nach Stadium auf unterschiedliche Argumentationsstrategien zurückgegriffen werden muss. Im Kontext einer beginnenden Demenz ist das liberale, autonomiebasierte Verständnis von Privatheit weiterhin in Anschlag zu bringen, da die Betroffenen in der Regel fähig sind, die eigenen Angelegenheiten selbstbestimmt zu verwalten. Im Zuge der fortschreitenden Beeinträchtigung kognitiver Fähigkeiten wie des Sprach- und Urteilsvermögens in mittleren Stadien gerät der autonomiebasierte Begriff von Privatheit allerdings zunehmend an Grenzen. Dennoch lässt sich anhand eines Spektrums verbaler und nonverbaler Äußerungen auch weiterhin ein Interesse von MmD an den verschiedenen Aspekten von Privatheit identifizieren, das es im Sinne ihres subjektiven Wohlergehens und verkörperten Selbst zu beachten und zu schützen gilt. Allerdings ist es für Außenstehende zunehmend schwierig, die Privatheitspräferenzen Betroffener einzuschätzen und gegen den erhöhten Betreuungs- und Schutzbedarf abzuwägen. In weit fortgeschrittenen Stadien demenzieller Erkrankungen muss daher auch auf andere Arten der Begründung der moralischen Bedeutung von Privatheit zurückgegriffen werden. Eine Möglichkeit bieten objektivistische Argumentationsstrategien, die die Bedeutung von Privatheit für Menschen mit weit fortgeschrittener Demenz unabhängig von der Perspektive der Betroffenen selbst verständlich zu machen suchen, etwa durch Rekurs auf ihre Würde oder ihr Wohlergehen. Die damit einhergehenden Begründungslasten mögen erheblich sein, sollten jedoch im Interesse einer weiteren Plausibilisierung des Privatheitsschutzes für Menschen auch mit fortgeschrittener Demenz nicht gescheut werden. Gerade objektive Vorstellungen eines guten Lebens, die z. B. auf grundlegende menschliche Befähigungen abzielen, ließen sich in

dieser Hinsicht für den Kontext der Demenz argumentativ fruchtbar machen, indem Privatheit z. B. als Grundlage für die Befähigung ausgewiesen wird, den Betroffenen zuträgliche Sorgebeziehungen aufrecht zu erhalten.

Vor diesem Hintergrund bedarf es weiterer begrifflicher, empirischer und normativer Forschungsanstrengungen, um die Bedeutung von Privatheit für MmD besser verstehen und berücksichtigen zu können. Dabei wären zunächst die Betroffenen selbst eingehender und differenzierter danach zu befragen, welche Empfindungen, Bedürfnisse und moralischen Anliegen das Konzept für sie berührt. Auf Grund ihrer Engführung mit dem Autonomiegedanken erscheinen die vorherrschenden theoretischen Konzeptionen von Privatheit für eine solche sozialempirische Exploration allerdings gerade mit Blick auf fortgeschrittene Krankheitsstadien kaum geeignet. Bevor sich der Wert des Privaten für MmD also in Form von empirischen Studien näher untersuchen lässt, muss zunächst ein erweitertes Verständnis des Privaten und seiner verschiedenen begrifflichen Implikationen, Konnotationen und Bezüge zu verwandten Begriffen wie dem der Nähe, Intimität oder Geborgenheit entwickelt werden. Hierfür kann sowohl auf die mit Privatheit assoziierten Werte und Funktionen zurückgegriffen werden als auch auf etablierte Kriterien in der Pflege von MmD. Durch die Verschränkung dieser begrifflich-philosophischen Analyse mit empirischer Exploration, etwa in Form qualitativer Sozialforschung mit Betroffenen und Angehörigen, kann in der Folge ein empirisch informierter Begriff von Privatheit entwickelt werden. Ein solcher Begriff hätte mindestens zwei Vorteile: Er böte zunächst eine Grundlage, um die Bedeutung und den Wert von Privatheit für MmD systematisch plausibel zu machen. Dies könnte nicht zuletzt einen Beitrag zur Sensibilisierung der Pflege für privatheitsrelevantes Verhalten von MmD leisten. Auch wer nicht mehr in der Lage ist, selbstbestimmt zu entscheiden, wem er Zutritt zum eigenen Zimmer gewähren will, wie die eingangs angesprochene Pflegeheimbewohnerin, kann durchaus ein nachvollziehbares Interesse an Schutz, Geborgenheit und vertrauten Nahbeziehungen – kurz: an Privatheit – haben, das es zu respektieren gilt. Darüber hinaus könnte eine in der empirisch informierten ethischen Auseinandersetzung mit Demenz entwickelte Perspektive allerdings auch dazu beitragen, die Engführung der Privatheitsdebatte mit dem liberalen Prinzip der individuellen Selbstbestimmung zu überwinden und so den Facettenreichtum und die moralische Vielschichtigkeit der Idee der Privatheit auch für Menschen ohne Demenz zu vergegenwärtigen.

Danksagung Wir danken Johannes Welsch (Göttingen) und Niklas Ellerich-Groppe (Oldenburg) für wichtige Anregungen.

Förderung Dieser Beitrag entstand im Kontext des BMBF-geförderten Projekts „EIDEC“ (Förderkennzeichen: 01GP1901B).

Funding Open Access funding enabled and organized by Projekt DEAL.

Einhaltung ethischer Richtlinien

Interessenkonflikt M. Schweda ist Mitglied des Beirats der Zeitschrift Ethik in der Medizin. E. Buhr gibt an, dass kein Interessenkonflikt besteht.

Ethische Standards Für diesen Beitrag wurden von den Autor/-innen keine Studien an Menschen oder Tieren durchgeführt. Für die aufgeführten Studien gelten die jeweils dort angegebenen ethischen Richtlinien.

Open Access Dieser Artikel wird unter der Creative Commons Namensnennung 4.0 International Lizenz veröffentlicht, welche die Nutzung, Vervielfältigung, Bearbeitung, Verbreitung und Wiedergabe in jeglichem Medium und Format erlaubt, sofern Sie den/die ursprünglichen Autor(en) und die Quelle ordnungsgemäß nennen, einen Link zur Creative Commons Lizenz beifügen und angeben, ob Änderungen vorgenommen wurden.

Die in diesem Artikel enthaltenen Bilder und sonstiges Drittmaterial unterliegen ebenfalls der genannten Creative Commons Lizenz, sofern sich aus der Abbildungslegende nichts anderes ergibt. Sofern das betreffende Material nicht unter der genannten Creative Commons Lizenz steht und die betreffende Handlung nicht nach gesetzlichen Vorschriften erlaubt ist, ist für die oben aufgeführten Weiterverwendungen des Materials die Einwilligung des jeweiligen Rechteinhabers einzuholen.

Weitere Details zur Lizenz entnehmen Sie bitte der Lizenzinformation auf http://creativecommons.org/licenses/by/4.0/deed.de.

Literatur

Alkhatib S, Waycott J, Buchanan G (2019) Privacy in aged care monitoring devices (ACMD): the developers' perspective. Stud Health Technol Inform 266:7–12. https://doi.org/10.3233/SHTI190765

Alkhatib S, Kelly R, Waycott J, Buchanan G, Grobler M, Wang S (2021) "Who wants to know all this stuff?!": understanding older adults' privacy concerns in aged care monitoring devices. Interact Comput 33(5):481–498. https://doi.org/10.1093/iwc/iwab029

Allen A (1988) Uneasy access. Privacy for women in a free society. Rowman & Littlefield, Totowa

Allen A (2014) Privacy in health care. In: Jennings B (Hrsg) Encyclopedia of bioethics, 4. Aufl. Macmillan, New York, S 2064–2073

Beard R, Fox P (2008) Resisting social disenfranchisement: negotiating collective identities and everyday life with memory loss. Soc Sci Med 66(7):1509–1520. https://doi.org/10.1016/j.socscimed.2007.12.024

Behr A, Meyer R, Holzhausen M, Kuhlmey A, Schenk L (2013) Die Intimsphäre. Z Gerontol Geriatr 46(7):639–644

Bloustein E (1964) Privacy as an aspect of human dignity. An answer to Dean Proser. NY Univ Law Rev 39(6):962–1007

Buhr E, Schweda M (2023) Technische Assistenzsysteme für Menschen mit Demenz: Zur ethischen Bedeutung von Beziehungen. In: Friedrich O, Seifert J, Schleidgen S (Hrsg) Mensch-Maschine-Interaktion – Konzeptionelle, soziale und ethische Implikationen neuer Mensch-Technik-Verhältnisse. mentis, Paderborn (im Erscheinen).

Buse C, Twigg J (2014) Women with dementia and their handbags: negotiating identity, privacy and 'home' through material culture. J Aging Stud 30:14–22. https://doi.org/10.1016/j.jaging.2014.03.002

Carlson E (2001) Videotaping to protect nursing facility residents: a legal analysis. JAMDA 2(1):41–44

Cipriani G, Ulivi M, Danti S, Lucetti C, Nuti A (2016) Sexual disinhibition and dementia. Psychogeriatrics 16(2):145–153. https://doi.org/10.1111/psyg.12143

Cohen J (2002) Regulating intimacy. A new legal paradigm. Princeton University Press, Princeton

Currie PM (2005) Balancing privacy protections with efficient research: Institutional Review Boards and the use of certificates of confidentiality. IRB Ethics Hum Res 27(5):7–12. https://doi.org/10.2307/3564529

DeCew J (1997) In pursuit of privacy. Law, ethics, and the rise of technology. Cornell University Press, Ithaca

Dekkers W (2011) Dwelling, house and home: towards a home-led perspective on dementia care. Med Health Care Philos 14(3):291–300. https://doi.org/10.1007/s11019-011-9307-2

Denke EM (2011) Stellvertretende Entscheidungen für Demenzkranke: mutmaßlicher Wille und andere determinierende Faktoren. Dissertation. Ludwig-Maximilians-Universität, München

Deutsche Gesellschaft für Fachkrankenpflege und Funktionsdienste (DGF) (2013) Ethische Prinzipien der Intensivpflegenden. https://www.dgf-online.de/wp-content/uploads/DGF-Ethik-Kodex_2014.pdf. Zugegriffen: 16. Dez. 2021
Deutscher Ethikrat (2012) Demenz und Selbstbestimmung. Stellungnahme, Deutscher Ethikrat, Berlin
Deutscher Ethikrat (2018) Hilfe durch Zwang? Professionelle Sorgebeziehungen im Spannungsfeld von Wohl und Selbstbestimmung. Stellungnahme. Deutscher Ethikrat, Berlin
Deutscher Ethikrat (2020) Robotik für gute Pflege. Stellungnahme. Deutscher Ethikrat, Berlin
Dichter M et al (2016) Die Lebensqualität von Menschen mit Demenz. Eine Metasynthese basierend auf den Aussagen von Menschen mit Demenz. In: Kovács L, Kipke R, Lutz R (Hrsg) Lebensqualität in der Medizin. Springer VS, Wiesbaden, S 287–302
Digby R, Bloomer M (2014) People with dementia and the hospital environment: the view of patients and family carers. Int J Older People Nurs 9(1):34–43. https://doi.org/10.1111/opn.12014
Dowideit A (2017) Wie viele Rechte bleiben einem Menschen im Pflegeheim? In: Die Welt, 23.11.2017. https://www.welt.de/vermischtes/plus170854328/Wie-viele-Rechte-bleiben-einem-Menschen-im-Pflegeheim.html. Zugegriffen: 5. Sept. 2022
Dresser R (1995) Dworkin on dementia: elegant theory, questionable policy. Hastings Cent Rep 25(6): 32–38. https://doi.org/10.2307/3527839
Dröes RM, Boelens-Van Der Knoop E, Bos J, Meihuizen L, Ettema T, Gerritsen D et al (2006) Quality of life in dementia in perspective. Dementia 5(4):533–558. https://doi.org/10.1177/1471301206069929
Dworkin R (1994) Life's dominion. An argument about abortion, euthanasia, and individual freedom. Vintage Books, New York
Dyck I, Kontos P, Angus J, McKeever P (2005) The home as a site for long-term care: meanings and management of bodies and spaces. Health Place 11(2):173–185. https://doi.org/10.1016/j.healthplace.2004.06.001
Eltis K (2005) Predicating dignity on autonomy? The need for further inquiry into the ethics of tagging and tracking dementia patients with GPS technology. Elder L J 13(2):387–415
Francis L, Francis J (2017) Privacy. What everyone needs to know. Oxford University Press, New York
Fried C (1968) Privacy. Yale L J 77(3):475. https://doi.org/10.2307/794941
Fuchs T (2018) Leiblichkeit und personale Identität in der Demenz. Dtsch Z Philos 66(1):48–61. https://doi.org/10.1515/dzph-2018-0005
Gavison R (1980) Privacy and the limits of law. Yale L J 89(3):421. https://doi.org/10.2307/795891
van der Geugten W, Goossensen A (2020) Dignifying and undignifying aspects of care for people with dementia: a narrative review. Scand J Caring Sci 34(4):818–838. https://doi.org/10.1111/scs.12791
Goffman E (1961) Asylums. Essays on the social situation of mental patients and other inmates. Anchor Books, New York
Graneheim UH, Norberg A, Jansson L (2001) Interaction relating to privacy, identity, autonomy and security. An observational study focusing on a woman with dementia and "behavioural disturbances", and on her care providers. J Adv Nurs 36(2):256–265. https://doi.org/10.1046/j.1365-2648.2001.01966.x
Herbst I (2021) Demenz und das Recht auf Nichtwissen: Die frühe Krankheitsphase zwischen Autonomie und Verantwortung. Brill mentis, Bielefeld
ICN (2014) ICN-Ethikkodex für Pflegende. https://www.wege-zur-pflege.de/fileadmin/daten/Pflege_Charta/Schulungsmaterial/Modul_5/Weiterfu%CC%88hrende_Materialien/M5-ICN-Ethikkodex-DBfK.pdf. Zugegriffen: 29. Apr. 2022
Immenschuh U (2018) Scham und Würde in der Pflege. GGP Fachz Geriatr Gerontol Pflege 2(3):115–119
Jacobsson A, Davidsson P (2015) Towards a model of privacy and security for smart homes. Proceedings of 2015 IEEE 2nd World Forum on Internet of Things (WF-IoT), Milan, 14.12.2015–16.12.2015, S 727–732
Jungert M (2018) „Ich habe mich sozusagen selbst verloren" – Biographische Identität, autobiographisches Gedächtnis und Alzheimer-Demenz. ZfPP 5(1):133–152. https://doi.org/10.22613/zfpp/5.1.6
Kittay E, Carlson L (Hrsg) (2010) Cognitive disability and its challenge to moral philosophy. John Wiley & Sons, Oxford
Klie T, Schuhmacher B, Denkinger H, Heine Y, Hils A (2010) Privatsphäre, (sozialer) Raum und personale Würde in Pflegeoasen. Vortrag, Gemeinsamer Kongress der DGGG/SGG, Berlin, 17. Sept. 2010 (https://agp-freiburg.de/downloads/Pflegeoase_Privatsphaere_DGGG2010_BS_Druck.pdf. Zugegriffen: 28. April 2022)
Knoll B, Kremer A, Reitinger E, Pichler B, Egger B (2017) Wenn die Orientierung versagt – unterwegs mit Menschen mit Demenz. REAL CORP 2017–PANTA RHEI–A World in Constant Motion. In: Proceedings of 22nd International Conference on Urban Planning, Regional Development and Information Society, S 647–657

Lillehammer H (2012) Autonomy, value, and the first person. In: Radoilska L (Hrsg) Autonomy and mental disorder. Oxford University Press, Oxford, S 192–214
Lind S (2005) Gemeinsame Sorge – geteilte Sorge: Zur Kommunikation zwischen Pflegekräften und Angehörigen von Demenzkranken. Pflegeimpuls 5(6):162–166
McColgan G (2005) A place to sit. J Contemp Ethnogr 34(4):410–433. https://doi.org/10.1177/0891241605275574
Moore A (2003) Privacy: its meaning and value. Am Philos Q 40(3):215–227
O'Brolcháin F, Gordijn B (2019) Privacy challenges in smart homes for people with dementia and people with intellectual disabilities. Ethics Inf Technol 21(3):253–265. https://doi.org/10.1007/s10676-019-09507-0
Oesterreich R (1981) Handlungsregulation und Kontrolle. Urban & Schwarzenberg, München
Pirhonen J, Pietilä I (2015) Patient, resident, or person: recognition and the continuity of self in long-term care for older people. J Aging Stud 35:95–103. https://doi.org/10.1016/j.jaging.2015.05.004
Reiman J (1976) Privacy, intimacy and personhood. Philos Public Aff 6(1):26–44
Rosenfeld L, Torous J, Vahia I (2017) Data security and privacy in apps for dementia: an analysis of existing privacy policies. Am J Geriatr Psychiatry 25(8):873–877. https://doi.org/10.1016/j.jagp.2017.04.009
Rössler B (2001) Der Wert des Privaten. Suhrkamp, Frankfurt am Main
Schell W (2011) Privatsphäre im Pflegeheim. Heilberufe 63(3):46–47
Schmidhuber M (2013) Der Stellenwert von Autonomie für ein gutes Leben Demenzbetroffener. Salzburger Beiträge zur Sozialethik, Bd. 5
Schoeman FD (1992) Privacy and social freedom. Cambridge University Press, Cambridge
Schües C (2020) Capturing space. Aging, (dis-)placement, or making room. In: Schweda M, Coors M, Bozzaro C (Hrsg) Aging and human nature, Springer, Cham, S 97–109
Schweda M, Schicktanz S (2021) Ethische Aspekte co-intelligenter Assistenztechnologien in der Versorgung von Menschen mit Demenz. Psychiatr Prax 48(S 01):37–41. https://doi.org/10.1055/a-1369-3178
Schweda M, Kögel A et al (2018) Prediction and early detection of Alzheimer's dementia: professional disclosure practices and ethical attitudes. J Alzheimers Dis 62(1):145–155. https://doi.org/10.3233/JAD-170443
Shenk D, Kuwahara K, Zablotsky D (2004) Older women's attachments to their home and possessions. J Aging Stud 18(2):157–169. https://doi.org/10.1016/j.jaging.2004.01.006
Smebye K, Kirkevold M (2013) The influence of relationships on personhood in dementia care: a qualitative, hermeneutic study. BMC Nurs 12(1):1–13. https://doi.org/10.1186/1472-6955-12-29
Thiele F (2013) Grundlagen der Menschenwürde-Diskussion. Einführung. In: Joerden JC, Hilgendorf E, Thiele F (Hrsg) Menschenwürde und Medizin. Ein interdisziplinäres Handbuch. Duncker & Humblodt, Berlin, S 13–34
Trescher H (2015) Die Würde des Privaten. Zur Diskussion institutionalisierter Lebensbedingungen von Menschen mit kognitiver Beeinträchtigung. Behindertenpädagogik 54(2):136–153
Welsch J, Buhr E (2022) Privacy-sensitive empowerment. Towards an integrated ethical concept for technology-assisted care for people with dementia. In: Rubeis G, Hartmann KV, Primc N (Hrsg) Digitalisierung der Pflege. V&R, Göttingen, S 185–197
Welsh S, Hassiotis A, O'Mahoney G, Deahl M (2003) Big brother is watching you—the ethical implications of electronic surveillance measures in the elderly with dementia and in adults with learning difficulties. Aging Ment Health 7(5):372–375. https://doi.org/10.1080/1360786031000150658
Westin A (1967) Privacy and freedom. LG Publishing, New York
Zwijsen S, Niemeijer A, Hertogh CM (2011) Ethics of using assistive technology in the care for community-dwelling elderly people: an overview of the literature. Aging Ment Health 15(4):419–427. https://doi.org/10.1080/13607863.2010.543662
